# Monograph of Diseases of the Nose and Throat

**Published:** 1897-09

**Authors:** 


### BOOK NOTICES.

A Monograph of Diseases of the Nose and Throat. By George H. Quay, M. D., Professor of Rhinology and Larynology, in the Cleveland Medical College. Philadelphia: Boericke & Tafel, 1897. Price, cloth, $1.25 net; by mail, $1.33.

This book of over two hundred pages is a concise description of the various diseases of the throat with the method of treatment. This method is a mixture of homœopathic indications for remedies and of the newer methods of the old school as adjuvant treatment. The book is gotten up of nearly the same size and uniform in binding with Bell's latest edition of *Diarrhœa* and Douglass' *Tongue Repertory*, both of which have been reviewed in these pages. It is also supplied with quite a number of illustrations.

The adjuvant treatment forms a very large part of the therapeutics of the book. Thus, in acute nasal catarrh, the author recommends the inhaling of Ammonia and Menthol crystals. He recommends also a spray of Menthol and Cocaine, to be used only during the watery stage, and not after that, as he believes it will prolong the attack if applied when the discharge becomes thick. A number of good indications for homœopathic remedies are then given. In the chronic form he gives quite a number of douches, and follows with a number of indications for the homœopathic prescription of remedies. He also recommends galvano-cautery in the chronic form. He seems to have no misgivings as to the chance of complications growing out of this cauterizing method of treatment. The writer of this notice has seen lung complications result from it, and also extensive ulceration resulting in fistulæ opening upon the bridge of the nose, and the ulceration extending upon the skin of the face.

In hypertrophic rhinitis he recommends surgical treatment and the use of very caustic acids. He is, of course, very careful to note the necessary precautions to be taken. He also recommends for the hemorrhage arising from operation, Peroxide of Hydrogen (Marchand's) and also applications of Marchand's *Glycozone*.

Atrophic rhinitis is also treated with Marchand's Glycozone. Homœopathic indications for remedies are given, but he holds out very litle encouragement for success with them.

Deviation of the septum is fully treated, and yet without an excess of verbiage. It is due either to traumatism or inheritance, and it is corrected only by operative procedures.

The writer of this review has seen some remarkable instances of deviation due to inheritance, where several individuals, all belonging to different generations of the same family, invariably had the deviation. It was even apparent at a glauce, when looking into the patient's face, without any internal inspection.

In treating hay fever, while he gives the homœopathic indications for remedies—

and they are very meagre—he does not place much reliance upon them, but upon various applications to the nose. Various other diseases of the nose are briefly treated, and then the author proceeds to the consideration of the associated diseases of the throat. He does not, in any of these, confine himself to homœopathic remedies, but, besides the usual operative methods, gives various means of treatment with crude drugs applied to the surfaces. In the homœopathic indications for tonsilitis he omits Lycopodium. With this drug in potency we have produced the most brilliant cures. Abscesses have been aborted by it, and the whole attack wonderfully shortened. Of course, the special indications for Lycopodium must be present, else it is of no use.

In all these nose troubles, with due respect to the learning of the author, we think he does not lay stress enough upon the factor of inheritance acting through the chronic miasm of psora, nor upon the need of adherence to the homœopathic remedies. His treatment is too suggestive of suppression of appearances. Neither does he anywhere speak of these diseases, especially acute, chronic, hypertrophic, and atrophic rhinitis, being often dependent upon suppressed gonorrhœa. The writer hereof is well persuaded of the truth of this observation. We do not overlook the fact that the author admits that "there is evidently a close sympathy existing between the genital organs and the nasal erectile tissue or corpora cavernosa." In this we cordially agree with him, and we are persuaded that we have seen cases of chronic rhinitis or chronic coryza in men aggravated, or, it may be, caused by excessive continence or abstaining from sexual indulgence. Of course, the scrofulous miasm was back of it all.

Another observation the author might have made, and that is the decline of intellectuality in the presence of these nose affections: This decline, ranging from mere stupidity and "thick-headed" blundering to downright imbecility. Lord Dundreary, in the play of "Our American Cousin," is a vivid illustration. The conception of this character by the writer of that play shows close observation and a very creditable insight into the peculiarities of these nose troubles on the part of a layman.

